# Diurnal changes in choroidal optical coherence tomography angiography indices over 24 hours in healthy young adults

**DOI:** 10.1038/s41598-023-30433-1

**Published:** 2023-03-02

**Authors:** Barsha Lal, David Alonso-Caneiro, Scott A. Read, Andrew Carkeet

**Affiliations:** Centre for Vision and Eye Research, School of Optometry and Vision Science, Queensland University of Technology, Kelvin Grove, Brisbane, Australia

**Keywords:** Diagnostic markers, Retina

## Abstract

This prospective study investigated the magnitude and pattern of variation in choroidal optical coherence tomography angiography (OCT-A) indices every 4 h over 24 h in healthy young myopic (n = 24) and non-myopic (n = 20) adults. Choriocapillaris and deep choroid en-face images from macular OCT-A scans were analysed from each session to extract magnification-corrected vascular indices including choriocapillaris flow deficit number, size and density and deep choroid perfusion density in the sub-foveal, sub-parafoveal, and sub-perifoveal regions. Choroidal thickness was also obtained from structural OCT scans. Significant variations over 24 h (P < 0.05) were observed in most of the choroidal OCT-A indices excluding sub-perifoveal flow deficit number, with peaks observed between 2 to 6 AM. For myopes, peaks occurred significantly earlier (3–5 h), and the diurnal amplitude was significantly greater for sub-foveal flow deficit density (P = 0.02) and deep choroidal perfusion density (P = 0.03) compared with non-myopes. Choroidal thickness also showed significant diurnal changes (P < 0.05) with peaks between 2 to 4 AM. Significant correlations were found between diurnal amplitudes or acrophases of choroidal OCT-A indices and choroidal thickness, intraocular pressure, and systemic blood pressure. This provides the first comprehensive diurnal assessment of choroidal OCT-A indices over 24 h.

## Introduction

Imaging the choroidal vasculature at various depths is valuable for understanding the pathophysiology of diseases such as myopia, age-related macular degeneration, diabetic retinopathy, central serous chorioretinopathy and uveitis^1,2^. The advent of optical coherence tomography angiography (OCT-A), an in vivo high resolution imaging method, allows en-face visualization of the choriocapillaris and deep choroidal microvasculature with good agreement with histological findings^1,3,4^. Indices quantifying vascular characteristics including flow deficit number, area and density and perfusion area or density can be extracted from these images with high sensitivity and repeatability^5,6^. A flow deficit or void in a choriocapillaris en-face image represents the spaces between the capillaries (the area where blood flow is absent or cannot be detected), whereas perfusion represents the area where blood flow is present^5,7^. The limited lateral resolution of OCT-A images makes it challenging to visualize flat capillaries of the choriocapillaris vasculature network, therefore flow deficit characteristics are widely used to study the perfusion of the choriocapillaris^5^.

Diurnal variation in the ocular measurements such as axial length and choroidal thickness plays an important role in regulating the ocular growth. Disruptions in these diurnal rhythms are thought to be a potential risk factor for myopia development^8^. An understanding of diurnal variations is also crucial for the reliable monitoring of physiological and pathological changes over time^9^. Most studies of choroidal diurnal changes using OCT-A have not found any variation in the choriocapillaris or deep choroidal OCT-A indices^10–13^ except for Sarwar et al.^14^ who reported a higher choriocapillaris perfusion density in the morning (68.74 ± 4.80 %) compared to the evening (67.57 ± 5.41 %). Siegfried et al.^10^ also found a significantly higher deep choroidal perfusion density in the afternoon compared to morning. However, these studies^10–14^ only assessed OCT-A over a limited time-frame during the day between 7 AM and 8 PM. Iwase et al.^15^ studied diurnal variation in the choroidal blood flow over a longer period, between 6 AM and 12 midnight using laser speckle flowgraphy and reported that highest blood flow occurred at 6 PM. The diurnal changes in the choroidal blood flow were also related to systemic blood pressure and ocular perfusion pressure. In addition, the choroidal vascularity index derived from macular structural OCT scans has also been shown to exhibit significant daytime variations between 6 AM and 9 PM^16^ with highest readings at 6 AM and lowest at 3 PM.

It is well-established that choroidal thickness changes over 24 h with peak thickness occurring at night^17,18^. In addition, choroidal thickness, and its vascular density and choriocapillaris perfusion have been reported to be interrelated with each other^19–21^. Other parameters including axial length, retinal thickness, intraocular pressure (IOP) and systemic blood pressure also exhibit variation over 24 h and are related to the choroidal thickness^8,9,17,18,22,23^, so it is reasonable to expect variation in the choroidal OCT-A indices over 24 h. To the best of our knowledge, no previous study has explored variation in the choroidal OCT-A indices, over a full 24-h period. Furthermore, axial length and refractive error exhibit associations with choriocapillaris density, choroidal blood flow and ocular blood flow^24–29^. Thus, refractive status could affect diurnal variation in choroidal vascular indices, however, no previous studies on daily variations in choroidal OCT-A indices have compared their findings between refractive groups^10–14^.

This study, therefore, investigated the magnitude and pattern of diurnal variation over 24 h in choroidal OCT-A microvasculature indices for myopic and non-myopic young healthy participants. The choriocapillaris and deep choroidal en-face images were extracted from the scans. Custom image analysis was used to extract OCT-A indices including flow deficit number, area and density and perfusion density corrected for magnification^30^ in the sub-foveal, sub-parafoveal and sub-perifoveal regions. The relationships between diurnal variation in choroidal OCT-A indices and various ocular and systemic measurements were also investigated.

## Methods

### Participants and data collection protocol

This was a prospective observational study examining diurnal changes in choroidal perfusion indices using OCT-A. A series of OCT-A scans were captured for 44 young healthy participants (mean age 23.2 ± 4.1 years; 19 females and 25 males) including 24 myopes and 20 non-myopes. Measurements were taken every 4 h at 7 time points, over 24 h starting at approximately 9 AM at the QUT Health Clinics Optometry Clinic. Participants were allowed to leave the clinic between 9 AM and 11 PM and were encouraged to carry out their normal daily activities. They stayed in the clinic overnight (with bedding provided) with lights off between 11 PM and 7 AM and were encouraged to sleep. The participants were woken up by the investigator for the 1 AM and 5 AM measurements and the measurements were taken at once. A 10 min wash period that involved watching television at 4 m was provided prior to each measurement session (excluding 1 AM and 5 AM). The room illumination was lowered (~ 2 Lux) for the nighttime measurements to limit the impact on normal circadian rhythm^31^.

The measurements included macular OCT-A and OCT scans, systemic blood pressure, IOP, and biometry measurements. The ocular measurements were taken only for the right eye. A detailed description of the study participants and procedures has been provided elsewhere^23^. The study was approved by Queensland University of Technology Human Research Ethics Committee and written informed consent was obtained after providing clear explanation of the testing procedures. All participants were treated in accordance with the tenets of the declaration of Helsinki. All participants enrolled had best-corrected visual acuity of 6/7.5 or better, noncycloplegic refraction between ± 6.00 DS and astigmatism ≤ 2.00 DC, normal ophthalmological findings, normal sleep/wake cycle, and good sleep quality (confirmed with the Pittsburgh Sleep Quality Index questionnaire)^32^. The subjective noncycloplegic spherical equivalent refraction (SER) of the right eye was used to classify participants as being myopic (SER ≤ − 0.50 D; mean SER: − 2.41 ± 1.95 D [range: − 0.50 to − 7.00 D]) or non-myopic (SER > − 0.50 D; mean SER: + 0.11 ± 0.38 D [range: − 0.38 to + 1.25 D]).

### OCT-A and OCT scanning protocol, image analysis and outcome indices

**Figure 1:**
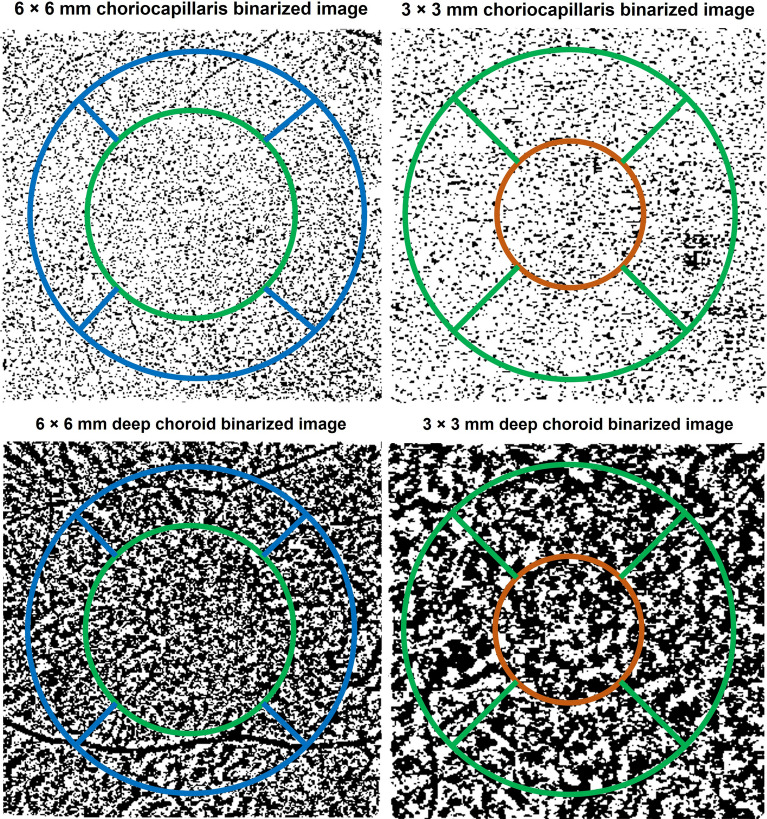
Example of the 6 × 6 mm (left) and 3 × 3 mm (right) binarized choriocapillaris (top) and deep choroidal (bottom) en-face image centred with modified early treatment diabetic retinopathy study (ETDRS) grid. Central/sub-foveal zone- inside orange circle, sub-parafoveal zone with quadrants- region between orange and green circles (in the left image) and sub-perifoveal zone with quadrants- region between green and blue circles (in the right image). The black pixels in the binarized choriocapillaris image represent the flow deficits. The black pixels in the binarized deep choroidal image represent the vasculature.

Two 3 × 3 mm and 6 × 6 mm macular angiography enhance depth imaging (EDI) scans were captured for the participant’s right eye in dim illumination using Zeiss OCT-A (AngioPlex software, version 11.0; Cirrus HD-OCT 5000, Carl Zeiss Meditec Inc, Dublin, California, USA). The Cirrus HD-OCT scans the eye at a rate of 68,000 A-scans per second with an axial resolution of 5 µm and a transverse resolution of 15 µm using a light source at 840 nm. AngioPlex OCT-A uses the OMAG^©^ algorithm to generate en-face angiographic images of the retina and choroid^33^. Previous studies have reported good repeatability in choroidal OCT-A indices across the sub-macular region^34,35^. Images were recaptured if signal strength was < 9, the images had severe motion artefacts, the image was not centred on the fovea or if they exhibited a horizontal scan tilt > 5 degrees^36^. The Zeiss automated segmentation demarcates the choriocapillaris layer extending from 29 µm beneath the retinal pigment epithelium (RPE) to 49 µm beneath the RPE and the deep choroidal layer is represented by a slab that extends from 64 to 115 µm below the RPE-fit line or Bruch’s membrane^33^. The en-face images of the choriocapillaris and deep choroid layer from the good quality 3 × 3 mm and 6 × 6 mm angiograms were exported for all the sessions for extraction of indices using custom image analysis software (Math-Works, Natick, MA, USA). The image analysis method is described in the Supplementary Section. The choriocapillaris and deep choroidal en-face images from the seven sessions were registered with each other to ensure that OCT-A indices were obtained from the same retinal region for each participant. A binary mask of the larger blood vessels from the projection artefacts of the overlying superficial retinal layer was generated and overlaid on the choroidal en-face images, to remove the influence of shadows of the larger blood vessels on the calculation of the OCT-A indices. Using a previously described schematic eye method incorporating individual biometry measures, a magnification correction factor was determined for each participant^30^. A modified ETDRS (early treatment diabetic retinopathy study) grid (Fig. 1) was then adjusted for magnification so that indices were obtained from the same sized retinal region between participants and then centred on the OCT-A en-face images. The modified ETDRS grid consisted of sub-foveal (1 mm annulus), sub-parafoveal (2.5 mm annulus) and perifoveal zones (5 mm annulus) (Fig. 1).

Three quantitative indices were extracted from the ETDRS zones of the binarized choriocapillaris images: flow deficit number, flow deficit size and flow deficit density^5,37^. The resolution of OCT-A is not sufficient to image the individual choriocapillaris vessels, so flow deficit characteristics were chosen. In a binarized choriocapillaris image, a flow deficit is displayed in black pixels where the blood flow is absent or cannot be detected (Fig. 1). An unconnected black pixel in the binarized choriocapillaris image is defined as a single flow deficit. Flow deficit number is defined as the total number of individually detected choriocapillaris flow deficits in the region of interest of the binarized image. Average flow deficit size (µm^2^) was the averaged size of all the detected flow deficits in the region of interest. Flow deficit number and size can together indicate a local or a global choriocapillaris change. A small number of larger flow deficits indicates a local choriocapillaris loss and a large number of small flow deficits indicates a scattered global loss^37^. Flow deficit density was calculated as the ratio of the image area occupied by the choriocapillaris flow deficits to the area of the region of interest in the binarized choriocapillaris image and expressed as a percentage.

As the average flow deficit size is calculated in µm^2^, this can be affected by image transverse magnification. Therefore, an additional transverse magnification correction^30^ (below equation) was used to compensate for this.

1 $$Corrected\; flow\; deficit\; size =Flow\; deficit\; size \times {Correction\; factor}^{2}$$

These corrected values were then used for further analysis. Flow deficit number and density are unitless, so these two indices did not require any further magnification correction.

The individual blood vessels in the deep choroid are larger in size compared to the choriocapillaris. This allows the visualization of the deep choroid blood vessels with OCT-A. Perfusion density was therefore extracted from the binarized deep choroidal images. Perfusion density was defined as the ratio of the image area occupied by the vasculature (displayed in black) to the total measured area in a binarized choroidal image and is calculated in percentage (Fig. 1). The perfusion density calculation considers both vessel length and diameter^38^. The deep choroidal flow appears dark as its intensity does not reach the threshold of the current OCT-A instruments due to the overlying RPE^39^, so the blood vessels here are indicated by black pixels in a binarized image as opposed to choriocapillaris layer where the blood vessels are displayed in white pixels. All the outcome indices were extracted for the sub-foveal and sub-parafoveal regions from the 3 mm scans and sub-perifoveal regions from the 6 mm scans (Fig. 1)^40–42^. The average of the outcome indices from the two choriocapillaris and deep choroidal images of each session were calculated.

At each measurement session, 5-line raster EDI scans were captured twice (horizontal foveal centered line of length 6 mm) using the Cirrus HD-OCT 5000 device. The choroidal layer was segmented and corrected for magnification and used to extract the choroidal thickness (between RPE and choroidoscleral interface) using previously described automated methods in MATLAB (Supplementary Section)^43^.

### Statistical analysis

Analysis was performed using SPSS (IBM Corp, Armonk, New York, USA), Microsoft Excel, and SigmaPlot (Systat Software, San Jose, CA). The measurements at each session were normally distributed (Kolmogorov–Smirnov test P > 0.05), whereas the diurnal amplitudes were not normally distributed (P < 0.05). A repeated measures analysis of variance (ANOVA) with post hoc Bonferroni pairwise adjustment was performed for each of the indices to investigate the time-of-day effect (within-subject factor), refractive group effect (between-subject factor) and their interactions in different ETDRS zones. For circular data, such as diurnal changes, analysis of variance might not detect phase differences in the presence of large inter-subject variation of amplitude, and also may not detect amplitude changes in the presence of significant inter-subject variation of phase. To look at these factors, circular statistics were used to analyse the diurnal data. Fourier analysis was used to determine acrophase (peak time) and amplitude of diurnal variation for each measurement and each participant independently^18,23,44^. In order to have good control of type 1 error rate, the Rayleigh test was used to assess dispersion of acrophase across the day in term of Rayleigh’s r value which ranges in value from 1 (all participants acrophases are identical) to 0 (participants acrophases are spread randomly across 24 h)^45,46^. Higher and significant Rayleigh’s r values represent more clustering of the acrophases. Mann–Whitney *U* test was used to assess whether there were differences in diurnal amplitudes between the refractive error groups and Watson’s *U*^2^ test was used to assess whether different refractive error groups had different acrophases.

Diurnal variation results for axial length, IOP, pulse pressure and systemic blood pressure for the participants have been provided elsewhere^23^ and were used to assess their associations with the diurnal variation in choroidal OCT-A indices. Pulse pressure was calculated as the difference between systolic and diastolic blood pressure (SBP and DBP). Mean arterial pressure (MAP) was calculated from the below formulae using (SBP and DBP).

$$MAP= \frac{1}{3} \times (SBP-DBP)+DBP$$

When parameters were compared, measurements were considered as in phase if their acrophases occurred at the same time (± 2 h) and antiphase if their acrophases occurred 12 h apart (± 2 h). Spearman’s Rho correlation (r_ρ_) and circular correlation (r_cc_) was performed to assess any relationship between the amplitudes and acrophases of different factors respectively.

## Results

Significant diurnal variations were found in most of the choriocapillaris and deep choroidal OCT-A indices in the sub-foveal, parafoveal and perifoveal zones. Table 1 shows the diurnal amplitudes, acrophase and statistical outcomes (Rayleigh test and repeated measures ANOVA outcomes) for the choriocapillaris and deep choroidal OCT-A indices for all participants. The diurnal findings of the choroidal OCT-A indices for all participants in each of the quadrants of the sub-parafoveal and perifoveal zones showed similar outcomes and are provided in Table S1 of the Supplementary Section. The daily mean for the choriocapillaris and deep choroidal OCT-A indices of the three regions and quadrants for all participants, myopes and non-myopes have been provided in the Supplementary Section Table S2.

**Table 1:** Daily mean, amplitude of diurnal variation, acrophase, Rayleigh test results and repeated measures ANOVA (analysis of variance) outcomes for the choriocapillaris and deep choroidal OCT-A indices of the sub-foveal, sub-parafoveal and sub-perifoveal regions for all participants. *Diurnal amplitude is also expressed as the percentage of the daily mean value. ^**†**^P values from repeated measures ANOVA for time of day, refractive error, and time by refractive error. P < 0.05 is highlighted in bold. *SD* standard deviation, *CD* circular deviation, *df* degree of freedom.

OCT-A indices	Sub-macular regions	Daily mean	Diurnal amplitude	Acrophase peak time	Rayleigh test	Repeated measures ANOVA		
						Time of day (df = 6, 258)	Time by refractive error (df = 6, 252)	Refractive error (df = 1, 42)
		Mean ± SD	Mean ± SD *(%)	Time ± CD (h)	Rayleigh’s r, P value	^†^P value		
Choriocapillaris-flow deficit number	Sub-fovea	272.33 ± 42.88	24.14 ± 15.13 (8.86%)	2:23 AM ± 4.20	**0.44, < 0.001**	**0.02**	0.99	**0.002**
Choriocapillaris-average flow deficit size (µm^2^)		399.26 ± 189.52	71.55 ± 80.67 (17.89%)	2:52 AM ± 4.50	**0.31, 0.010**	**0.04**	0.06	**0.009**
Choriocapillaris-flow deficit density (%)		14.15 ± 6.72	2.64 ± 1.68 (18.66%)	3:00 AM ± 4.00	**0.46, < 0.001**	**< 0.001**	**0.02**	**0.001**
Deep choroidal-perfusion density (%)		57.22 ± 4.41	1.53 ± 0.79 (2.67%)	4:48 AM ± 4.54	**0.29, 0.034**	0.06	0.41	0.57
Choriocapillaris-flow deficit number	Sub-parafovea	1446.76 ± 220.77	108.66 ± 71.79 (7.51%)	2:19 AM ± 4.19	**0.40, < 0.001**	**0.006**	0.92	**0.001**
Choriocapillaris-average flow deficit size (µm^2^)		374.05 ± 172.29	51.85 ± 55.97 (13.86%)	2:23 AM ± 4.29	**0.37, 0.001**	0.08	**0.04**	**0.001**
Choriocapillaris-flow deficit density (%)		13.50 ± 6.44	2.56 ± 3.97 (18.96%)	4:33 AM ± 4.69	**0.46, < 0.001**	**0.002**	**0.03**	**0.001**
Deep choroidal-perfusion density (%)		53.98 ± 3.68	1.51 ± 0.91 (2.80%)	5:05 AM ± 4.29	**0.37, 0.003**	**0.02**	**0.03**	0.35
Choriocapillaris-flow deficit number	Sub-perifovea	2499.66 ± 426.47	227.03 ± 127.86 (9.08%)	4:43 AM ± 4.73	0.23, 0.090	0.08	0.54	**< 0.001**
Choriocapillaris-average flow deficit size (µm^2^)		608.53 ± 275.43	98.66 ± 105.02 (16.21%)	6:13 AM ± 4.78	0.22, 0.090	0.11	0.38	**0.001**
Choriocapillaris-flow deficit density (%)		10.70 ± 5.92	2.39 ± 2.11 (22.34%)	4:25 AM ± 4.65	**0.27, 0.048**	**0.03**	0.48	**0.002**
Deep choroidal-perfusion density (%)		51.57 ± 2.79	1.45 ± 0.93 (2.81%)	3:12 AM ± 4.32	**0.36, 0.003**	**< 0.001**	0.20	0.21

### Diurnal variation in choriocapillaris OCT-A indices for all participants

Significant diurnal variations over 24 h (Table 1) were observed in the sub-foveal choriocapillaris flow deficit number (P = 0.02), flow deficit size (P = 0.046) and flow deficit density (P < 0.001) with respective diurnal amplitude of 24.14 ± 15.13, 71.55 ± 80.67 µm^2^ and 2.64 ± 1.68 %. In the sub-parafoveal region, choriocapillaris flow deficit number (P = 0.006) and flow deficit density (P = 0.002) demonstrated significant diurnal variation with respective diurnal amplitude of 108.66 ± 71.79 and 2.56 ± 3.97 %. In the sub-perifoveal region, significant diurnal variation was noted only in the choriocapillaris flow deficit density (P = 0.03) with diurnal amplitude of 2.39 ± 2.11 %.

Bonferroni adjusted pairwise comparisons revealed that the sub-foveal flow deficit density at 9 AM (13.47 ± 6.42 %) was significantly lower than the flow deficit density at 1 AM (14.79 ± 7.27 %; P = 0.01) and 5 AM (14.95 ± 6.81 %; P = 0.04). Sub-parafoveal flow deficit density at 9 AM (12.94 ± 6.08 %) was also significantly lower than the flow deficit density at 1 AM (14.13 ± 7.05 %; P = 0.04).

**Figure 2:**
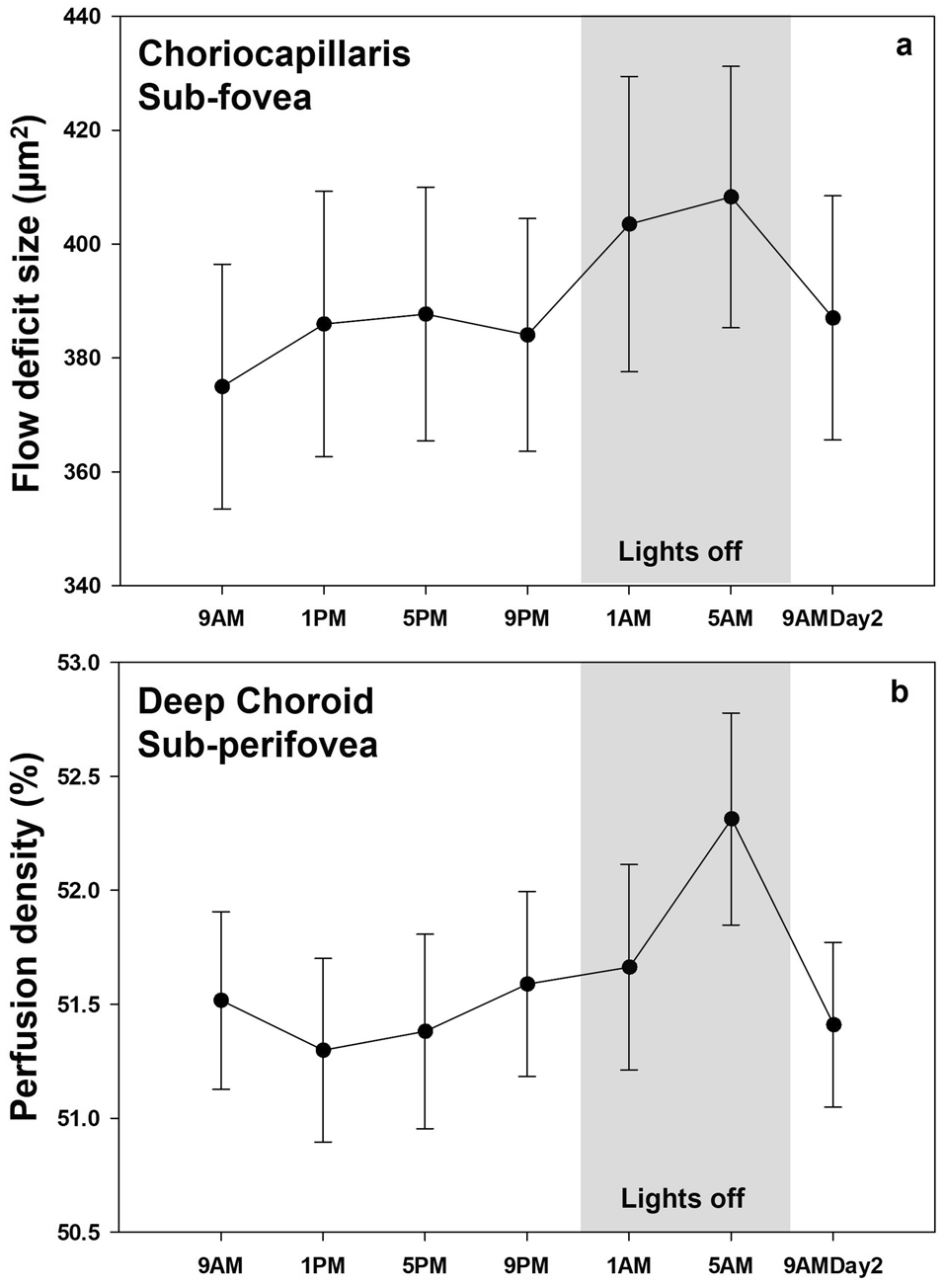
Choriocapillaris flow deficit indices (**a**) and deep choroid perfusion density (**b**) plotted across 24 h for all participants in the three regions. The peak for these indices were observed between 2 and 6 AM. For all time points, error bars indicate one standard error of the mean.

The Rayleigh test showed significant clustering of acrophases (i.e., significant diurnal variation) in all the choriocapillaris OCT-A indices for all the three regions (all P < 0.05) except sub-perifoveal flow deficit number and size (P = 0.09 for both) (Table 1). The peaks for the three choriocapillaris indices were observed between 2 and 6 AM in the three regions (Table 1). Figure 2a plots the changes in the choriocapillaris flow deficit indices across 24 h to demonstrate the general diurnal pattern for all participants.

### Diurnal variation in deep choroidal perfusion density for all participants

The deep choroidal perfusion density exhibited significant diurnal variation in the sub-parafoveal and sub-perifoveal regions (P < 0.001 for both) with diurnal amplitudes of 1.51 ± 0.91 % and 1.45 ± 0.93 % and acrophases at 5:05 AM and 3:12 AM (Table 1). The sub-foveal deep choroidal perfusion density did not exhibit significant diurnal variations. Bonferroni adjusted pairwise comparisons revealed significantly higher perfusion density (P = 0.049) at 5 AM (54.39 ± 3.88 %) than 5 PM (53.70 ± 3.72 %) in the sub-parafoveal region. The perifoveal perfusion density measurements at 5 AM (52.29 ± 3.04 %) were also significantly higher than measurements at 9 AM (51.45 ± 2.64 %; P = 0.02), 1 PM (51.26 ± 2.67 %; P = 0.002) and 5 PM (51.32 ± 2.86 %; P = 0.002). The Rayleigh test revealed significant clustering of acrophases (Table 1) (all P < 0.05) for perfusion density in all the three regions indicating significant diurnal variations. Figure 2b illustrates an example pattern of the change in deep choroid perfusion density across 24 h for all participants in the perifoveal region.

### Time by refractive group interaction for choriocapillaris OCT-A indices

**Figure 3:**
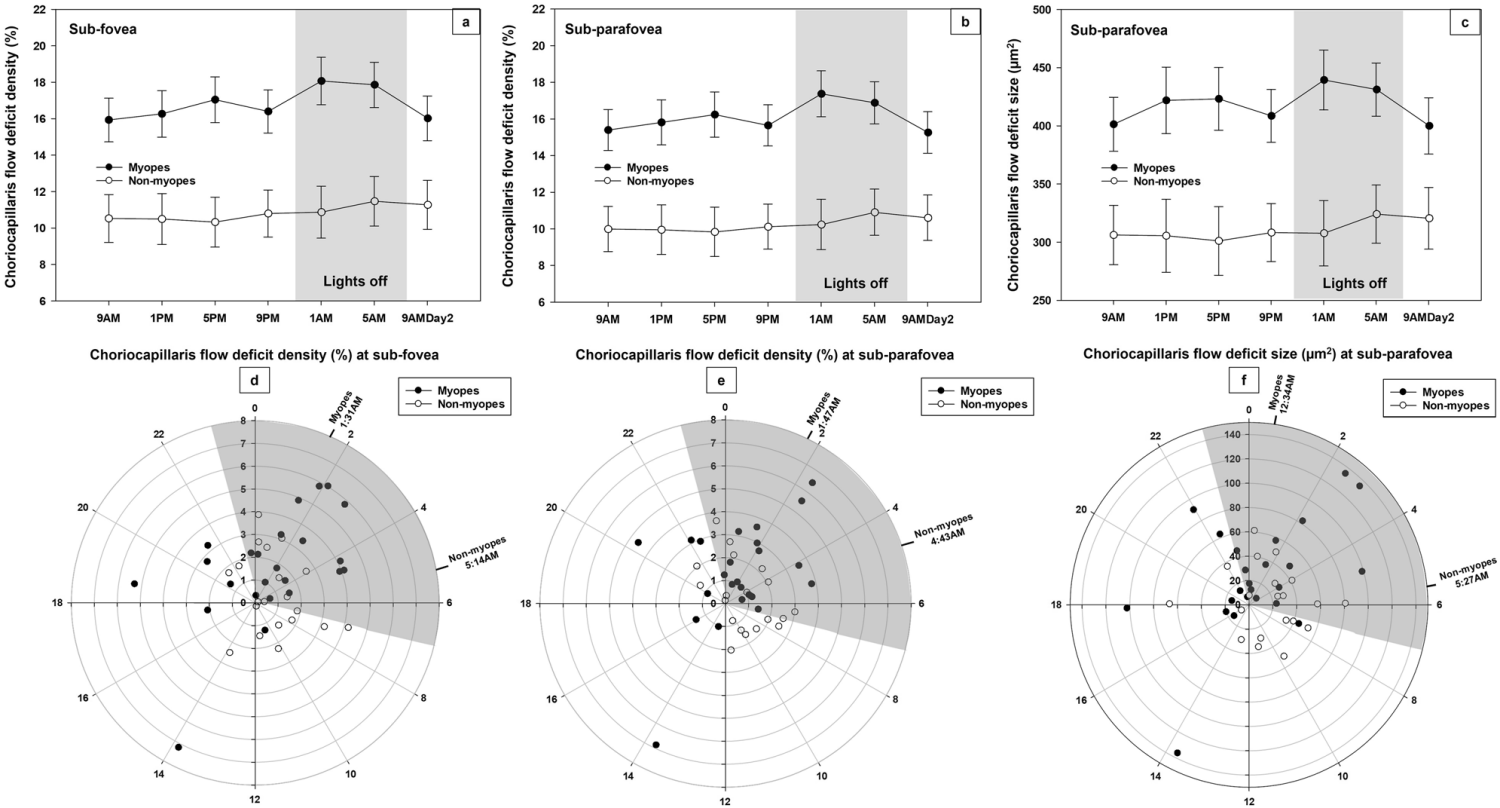
The top images (**a**–**c**) plots, across 24 h, sub-foveal and sub-parafoveal choriocapillaris flow deficit density and sub-parafoveal choriocapillaris flow deficit size in myopes and non-myopes. Significant time by refractive group interactions (P < 0.05) were found in the three indices with myopes and non-myopes demonstrating different pattern of variation. For all time points, error bars indicates one standard error of the mean. The bottom images (**d**–**f**) shows polar diagrams for sub-foveal and sub-parafoveal choriocapillaris flow deficit density and sub-parafoveal choriocapillaris flow deficit size depicting diurnal amplitude of change (distances from center) plotted against acrophase time (around the perimeter, 24-h clock) in myopes and non-myopes.

Myopes differed from non-myopes in the pattern of their diurnal variation in the sub-foveal (P = 0.02) and sub-parafoveal choriocapillaris flow deficit density (P = 0.03) (Fig. 3a,b). Further analysis showed that the acrophase of the choriocapillaris flow deficit density in the sub-foveal and sub-parafoveal regions occurred significantly earlier (P = 0.04) in myopes (1:31 AM ± 3.58 h and 1:47 AM ± 3.64 h) compared to non-myopes (5:14 AM ± 3.97 h and 4:43 AM ± 4.05 h) (Fig. 3d,e). In addition, the diurnal amplitude of sub-foveal flow deficit density was also noted to be significantly higher (P = 0.02) in myopes (3.16 ± 1.92%) than non-myopes (2.02 ± 1.10 %). Similarly, the sub-parafoveal choriocapillaris flow deficit size also demonstrated a significantly different acrophase time (P = 0.04) between the refractive groups with acrophases occurring significantly earlier (P = 0.04) in myopes (12:34 AM ± 4.79 h) than non-myopes (5:27 AM ± 4.22 h) (Fig. 3c,f). However, diurnal amplitudes were not significantly different between the refractive groups.

### Time by refractive group interaction in the deep choroid OCT-A indices

**Figure 4:**
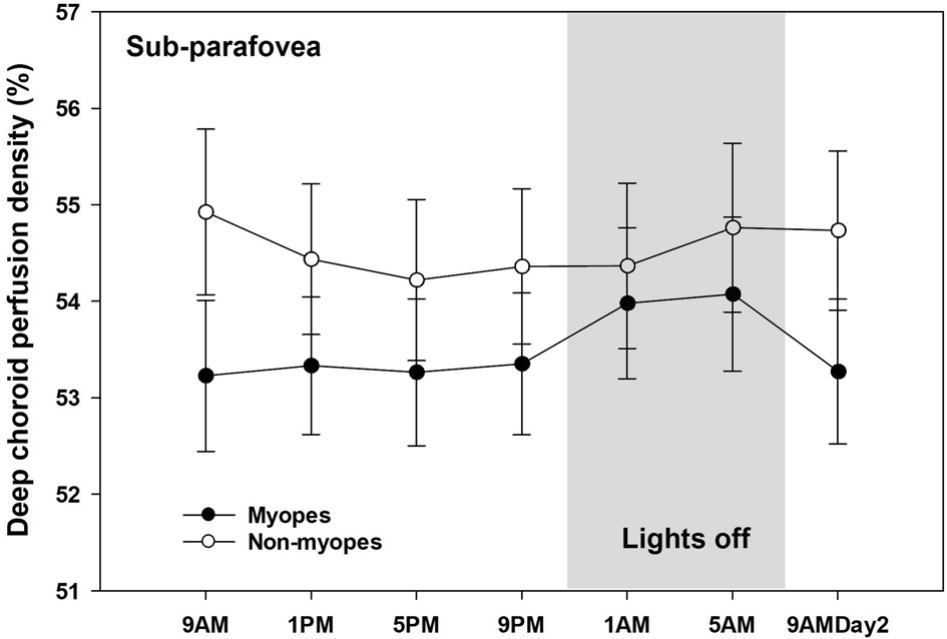
Sub-parafoveal deep choroid perfusion density in myopes and non-myopes plotted across 24 h. A significant time by refractive group interaction for both indices (P = 0.03) was noted with myopes and non-myopes demonstrating different pattern of variation, however their acrophase difference did not reach statistical significance. Error bars indicates one standard error of the mean for all time points.

A significant interaction between time and refractive group (P = 0.03) was noted in the sub-parafoveal deep choroidal perfusion density (Fig. 4). The diurnal amplitude of the sub-foveal deep choroidal perfusion density was significantly (P = 0.03) higher in myopes (1.76 ± 0.90 %) than non-myopes (1.25 ± 0.53 %). However, the acrophases were not significantly different between the refractive groups.

All of the choriocapillaris OCT-A indices in the three zones and quadrants showed a significant refractive group effect (Table 1 and Table S1) with the daily mean being significantly higher in myopes than non-myopes whereas the deep choroidal perfusion density did not exhibit any significant refractive group effects in any of the zones (Supplementary Table S2).

### Diurnal variation in the choroidal thickness

Significant variation over 24 h was found in the choroidal thickness of foveal (P = 0.03), parafoveal (P = 0.02) and perifoveal zones (P = 0.001) with the diurnal amplitude being 23.98 ± 14.42 µm, 17.36 ± 9.52 µm and 17.76 ± 11.06 µm respectively. The choroidal thickness acrophases occurred between 2 to 4 AM for all the three zones. However significant clustering of the acrophases was noted only for foveal and perifoveal choroidal thickness (Rayleigh’s r = 0.42; P < 0.001 and Rayleigh’s r = 0.34; P = 0.009). The choroidal thickness did not exhibit any significant interaction between time and refractive group (P > 0.05).

### Associations of the choriocapillaris and deep choroidal OCT-A indices

**Figure 5:**
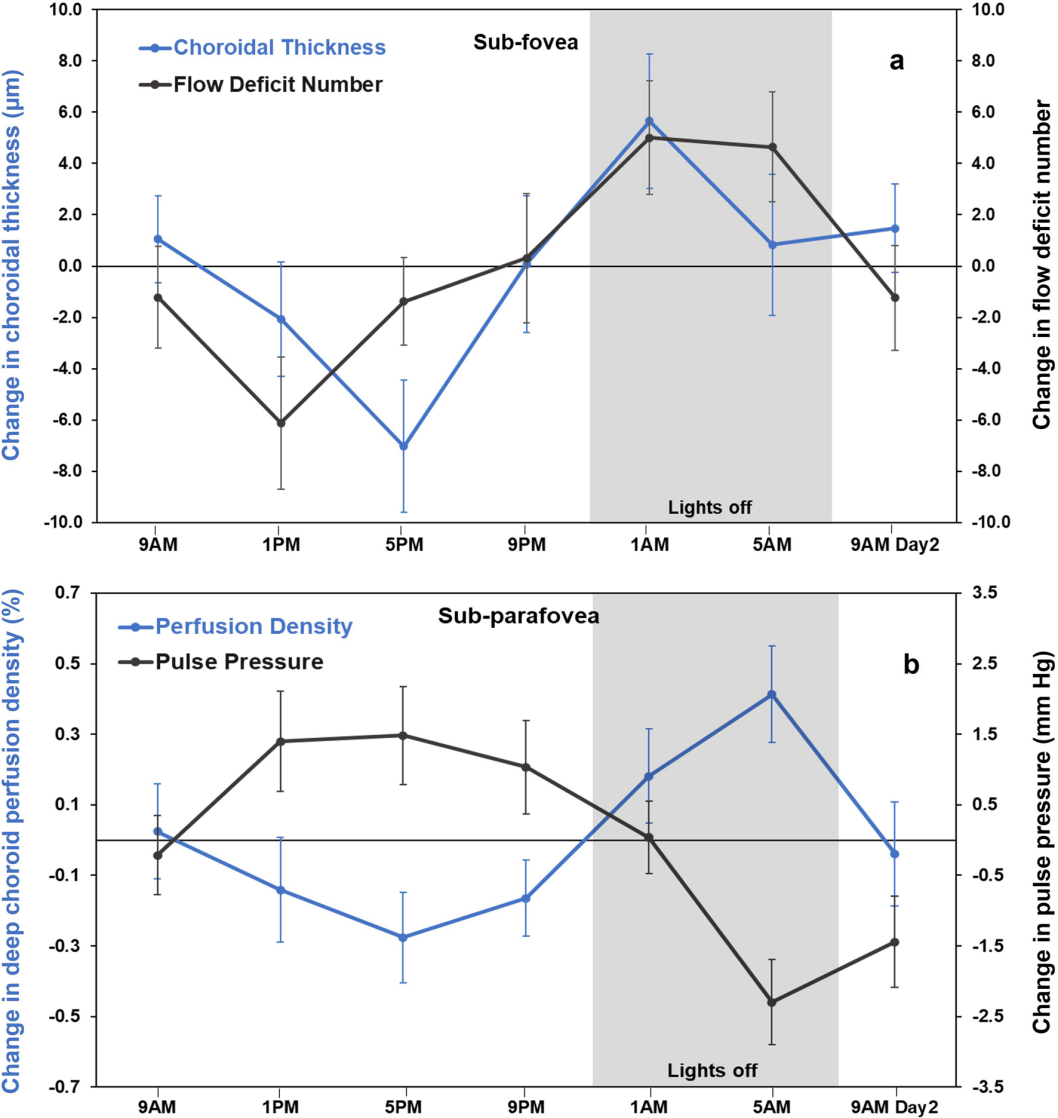
(**a**) Mean change in choroidal thickness (blue) and choriocapillaris flow deficit number (black) in the foveal region across 24 h demonstrating their variations in approximately in phase with each other. (**b**) Mean change in perfusion density of the deep choroid (blue) and pulse pressure (black) in the parafoveal region across 24 h demonstrating their variation in approximately antiphase with each other. For all time points, error bars indicate one standard error of the mean.

The diurnal amplitudes of sub-parafoveal choroidal thickness were significantly negatively correlated with the diurnal amplitude of sub-parafoveal flow deficit size (r_ρ_ = − 0.37; P = 0.01) and flow deficit density (r_ρ_ = − 0.38; P = 0.01). The sub-foveal flow deficit number acrophase was also positively correlated with the acrophases of choroidal thickness in three regions (sub-foveal: r_cc_ = 0.37; P = 0.005, sub-parafoveal: r_cc_ = 0.33; P = 0.02 and sub-perifoveal: r_cc_ = 0.36; P = 0.006) (Fig. 5a).

The diurnal amplitude of sub-foveal deep choroidal perfusion density was negatively correlated with diurnal amplitude of IOP (r_ρ_ = − 0.35; P = 0.028). The acrophases of the flow deficit measurements in the three regions were also negatively correlated with the acrophases of IOP (r_cc_ = − 0.29 to − 0.47; P < 0.001).

Both sub-foveal and sub-parafoveal flow deficit number diurnal amplitudes were negatively correlated with MAP (r_ρ_ = − 0.32; P = 0.046 for both). The acrophase of pulse pressure were negatively correlated with the acrophases of the flow deficit number and deep choroid perfusion density in the three regions (r_cc_ = − 0.29 to − 0.32; P < 0.05) (Fig. 5b).

## Discussion

This study is the first to report the pattern and magnitude of diurnal variation in the macular choriocapillaris and deep choroidal OCT-A indices over 24 h among young healthy adults and the first to compare such findings between myopes and non-myopes. The choriocapillaris OCT-A indices including sub-foveal and parafoveal flow deficit number, sub-foveal flow deficit size and flow deficit density demonstrated significant diurnal variations. Significant diurnal variation was also observed in deep choroidal perfusion density for all three regions. The acrophase for these choriocapillaris and deep choroidal indices were observed between 2 and 6 AM. The acrophases occurred approximately 3 to 5 h earlier in myopes compared to non-myopes in sub-foveal flow deficit density and sub-parafoveal choriocapillaris flow deficit density and size and deep choroidal perfusion density. The myopes also showed significantly higher diurnal amplitudes of sub-foveal flow deficit density and deep choroidal perfusion density than non-myopes.

The choriocapillaris represents the inner part of the choroid whereas Sattler’s and Haller’s layers represents the outer or deeper part of choroid^47^. Previous studies have examined the daytime variation in choriocapillaris or deep choroidal perfusion over a limited duration of 11–13 h among healthy participants^10–14^. They used different indices, different OCT devices, recruited participant in different ages and captured images using 3 × 3 mm scanning protocols to investigate diurnal variation compared to the present study. Lin et al.^12^ did not find any significant observable diurnal variation in flow deficit characteristics between 9 AM to 5 PM in healthy adults. Similarly, other studies did not report any significant diurnal changes during the day in choriocapillaris perfusion/vascular area or density^10,11^ except for Sarwar et al.^14^ who found significantly higher choriocapillaris perfusion density (which represents the ratio of the image area occupied by blood vessels to the total image area) at 9 AM compared to 6 PM among healthy adults. A previous study also evaluated the deep choroidal sublayer (Haller’s and Sattler’s layer) perfusion using OCT-A between 7 AM to 8 PM and found significant changes with a peak observed in afternoon^10^. The present study evaluated the choriocapillaris and deep choroidal OCT-A indices every 4 h over 24 h. The flow deficit characteristics and deep choroidal perfusion density were observed to be highest between 2 and 6 AM. This indicates lowest choriocapillaris perfusion and highest deep choroidal perfusion at night throughout the macular region. While in the dark, photoreceptors are extremely active and most of their oxygen comes from the choroidal circulation^48^, which may relate to our findings of peak deep choroidal perfusion at night. A high deep choroidal perfusion density detected from the en-face OCT-A image indicates an increase in the length and diameter of the deep choroidal blood vessels^38^. It has been reported that choroidal vascular dilation and choriocapillaris perfusion are negatively associated in healthy adults possibly explaining the peak of flow deficit characteristics at night^7,49^.

Changes in choroidal blood flow have also been associated with posture changes among previous studies^7,50,51^. An increase in hydrostatic pressure in the supine position results in the expansion of the choroid. The choroidal blood flow increases by 11 % from standing to supine position mainly due to 8 % change in measured blood velocity^50^. It is speculated that this increase in choroidal blood flow is mostly due to the increase in perfusion pressure during posture change which may be mediated by constriction of the small choriocapillaris^50,52^. This may cause partial closure (and reduce the blood flow velocity below the threshold of the OCT-A device) or complete closure of the vessels, resulting in an increase in flow deficits^20,52^. The night-time (1 AM and 5 AM) measurements in the current study could also be influenced by the postural change as they were taken within ~ 5 min of participants waking up. The diurnal amplitude of the choriocapillaris flow deficit density in the current study ranged between 2 and 3 % (Table 1), higher than the findings reported by Sarwar et al.^14^ (1.17 % across 9 h) probably owing to the longer measurement duration in the present study.

Choroidal thickness is one of the major determinants of deep choroidal vascular density and index but has not been found to be associated with choriocapillaris indices^19,21,53–56^. Another study reported that the diurnal changes in the choroidal thickness were primarily a result of changes in the luminal areas of the choroid during the day^16^. Additionally, Sarwar et al.^14^ reported significant positive correlation between diurnal changes occurring in choroidal thickness and in choriocapillaris perfusion in the subfoveal region. In the present study, choroidal thickness variations were in phase with the deep choroidal perfusion density as well as choriocapillaris flow deficit indices throughout the macula (Fig. 5a) with their acrophases and amplitudes related. A review by Liu et al.^57^ suggests that increases in choroidal blood flow may be associated with an increase in choroidal thickness, supporting the current study findings.

The present study also demonstrates for the first time, significant differences in the pattern of the diurnal functions between myopes and non-myopes of choriocapillaris sub-foveal flow deficit density and sub-parafoveal flow deficit density and size and deep choroidal perfusion density. Their acrophases occurred significantly earlier in myopes than non-myopes. The choriocapillaris flow deficit indices have been reported to be significantly higher among myopes^28,58,59^. The choriocapillaris and deep choroidal layer tend to exhibit a decrease in density and blood flow in myopic eyes due to axial elongation, choroidal stretching and thinning, low pulsatile ocular blood flow, low ocular perfusion pressure and high vascular resistance index of the choroidal blood vessels and central retinal artery^24–26,60,61^. Among these factors, vascular resistance, ocular perfusion pressure, and axial length also changes significantly over 24 h^18,23,62,63^. The present study did not find any significant difference between refractive groups in the diurnal variation of axial length and ocular perfusion pressure. It is speculated that differences in diurnal variation in vascular resistance may underlie the refractive group differences observed. A previous study reported that myopes have different local choroidal vascular reactivity than emmetropes, possibly explaining the different diurnal variation between myopes and non-myopes in the present study^64^. We also found differences in both acrophase and amplitude for choriocapillaris flow deficits whereas the deep choroid only differed in amplitude between refractive groups. It can be speculated that the local vascular reactivity of choriocapillaris and deep choroid may be different, possibly explaining the different acrophase and amplitude findings. The differences in the diurnal pattern of choroidal vasculature between the two refractive groups were similar to the diurnal findings in superficial retinal vasculature as reported in our previous work^23^. In addition, the sub-foveal flow deficit density and deep choroidal perfusion density diurnal amplitudes were also greater in myopes than non-myopes in this study. This indicates that the myopes have more prominent daily circadian changes in choroidal microvasculature compared to non-myopes.

Studies have shown that changes in IOP can affect the blood flow pattern^65^ and flow deficit density^21^ of the choriocapillaris and the choroidal blood flow^66^. In the current study, IOP and sub-foveal deep choroidal perfusion density diurnal amplitudes were negatively correlated, similar to the previous cross-sectional study findings^66^. However, the IOP acrophase was negatively related to that of choriocapillaris flow deficit indices, contrary to earlier cross-sectional findings^21^ that higher IOP was associated with higher choriocapillaris flow deficits. This could be due to the inclusion of older adults between 31 to 78 years in the previous cross-sectional study as opposed to the young adults (18–35 years) in the present diurnal study. In the present study, the choriocapillaris flow deficit number and MAP diurnal amplitudes were negatively correlated. In addition, the pulse pressure variation was also in antiphase with the choriocapillaris flow deficit density and deep choroidal perfusion density variation (Fig. 5b). Small but significant increase in choroidal blood flow with increasing blood pressure have been observed in a study by Polak et al.^67^.

The choriocapillaris and choroidal perfusion play an integral role in the maintenance of normal ocular physiology as well as in the pathophysiology of various ocular conditions such as age-related macular degeneration, diabetic retinopathy, glaucoma, and myopia^1,3,68–71^. The findings from the current study in healthy young adults will help in differentiating between normal physiological diurnal variation and diurnal variation in pathological conditions of choriocapillaris and deep choroidal perfusion. A recent cross-sectional study by Cheng et al.^21^ reported an annual increase of 0.03% in the flow deficit density for the macular region among participants older than 30 years whereas Fujiwara et al.^19^ found an annual decrease of 0.1% in choroidal perfusion among participants aged between 6–80 years. As compared to this annual decrease of 0.1% in choroidal perfusion, the amplitudes of diurnal variation of choriocapillaris flow deficit and deep choroidal perfusion density diurnal amplitudes in the macular region in the current study are considerable (7–23% and 2–3% of the daily mean). These diurnal changes therefore need to be considered while defining a meaningful clinical change. The choroidal OCT-A indices measured at night (1 AM and 5 AM) were significantly different from the measurements during the day (between 9 AM and 9 PM). The variations in the OCT-A indices observed during the day (between 9 AM and 5 PM) were considerably smaller than the 24 h diurnal amplitude of the OCT-A indices. For instance, the changes in sub-foveal flow deficit density during the day was averaged 0.37% compared to the diurnal amplitude of 2.64%. Given this, it is safe to assume that clinical quantification of choroidal OCT-A indices is unlikely to be significantly affected by diurnal variation during the usual clinic hours between 9 AM and 5 PM.

Given that the current study examined young adults with a small range of ages, future studies can focus on exploring diurnal variation in the OCT-A indices over 24 h among healthy children and older adults. It is believed that diurnal variations in choroidal thickness is associated with ocular growth and myopia development^8,72^. It can be speculated that choroidal blood perfusion diurnal variations might show similar results. Although the current study reported differences in the pattern of diurnal variations between myopes and non-myopes, further research is also needed to understand whether these differences are a cause or a consequence of myopia, and to determine their relationship with axial eye growth. The present study did not include moderate and high hyperopes (> + 1.25 D). Previous studies have reported that diurnal variation in IOP and choroidal thickness are influenced by hyperopia^73,74^. It can be speculated that hyperopia may have similar effects on the diurnal variation of choroidal perfusion. Future studies can explore the association between hyperopia and diurnal variation in choroidal OCT-A indices. The current study used a spectral domain OCT-A with EDI with a wavelength of 840 nm for imaging the choroid and choriocapillaris. Other imaging methods such as swept source OCT-A uses 1060 nm wavelength and provides better visualization of the choriocapillaris and deeper choroid. Further studies can be planned to see if the magnitude of diurnal variation in the choriocapillaris and deep choroidal perfusion over 24 h is influenced by these imaging modalities.

To conclude, this study is the first to report significant diurnal variations in the macular choriocapillaris and deep choroidal OCT-A indices over 24 h. Peaks for these indices were observed between 2 and 6 AM. Myopes and non-myopes showed different acrophase and amplitude of diurnal variation in the choriocapillaris and deep choroidal OCT-A indices of the foveal and parafoveal regions. The amplitude and acrophase of the choriocapillaris and choroidal OCT-A indices demonstrated significant correlations with choriocapillaris, IOP, pulse pressure and MAP.

## Supplementary Information

Supplementary Information.

## Data Availability

The datasets generated and analysed during this study are available from the corresponding author on reasonable request.
