# Supplementary material for: Diurnal changes in choroidal optical coherence tomography angiography indices over 24 hours in healthy young adults
Source: Sci Rep. 2023 Mar 2;13:3551. doi: 10.1038/s41598-023-30433-1 (PMC9981752; doi:10.1038/s41598-023-30433-1)
Supplement: Supplementary file 1 — Supplementary Information. [file 41598_2023_30433_MOESM1_ESM.pdf]

Diurnal changes in choroidal optical coherence tomography angiography indices over 24 hours in healthy young adults

Barsha Lal, David Alonso-Caneiro, Scott A Read, Andrew Carkeet

Choriocapillaris and deep choroidal OCT-A *en-face* image analysis and extraction of indices: The exported choriocapillaris and deep choroidal *en face* images (.png) were analysed using custom image analysis developed with MATLAB (MathWorks, Natick, Massachusetts, USA) software.

Figure S1 summarizes the overview of the image analysis and process for extracting indices for the choriocapillaris image of 3 x 3 mm scan size. The choriocapillaris from the seven measurements time points were aligned to each other with respect to the information derived from their respective superficial retinal layer image compensating for image geometrical transformation (decentration, rotation and scaling) between them. This ensured that indices within subjects were derived from the same region of the choriocapillaris images. A binary mask of the larger blood vessels from the SRL images was also generated by applying a fixed threshold binarization algorithm.[1] This binary mask (corrected for transformation) was then overlaid on the choriocapillaris *en face* images to remove the influence of shadow of the larger blood vessels from the calculation of the choriocapillaris indices. These choriocapillaris images were then binarized using the local Phansalkar method (square window size: 7 pixels for 3 x 3 mm and 4 pixels for 6 x 6 mm).[2] A modified ETDRS grid with central (1 mm annulus) and sub-parafovea (2.5 mm annulus) was rotated and scaled to the average amount of rotation and magnification of the 7 sessions using previously described methods[3] and was placed on the choriocapillaris image of 3 x 3 mm scan size so that indices between subjects were derived from the same sized choriocapillaris regions. Similar steps were used for choriocapillaris images of 6 x 6 mm scans except that the modified ETDRS grid constructed here consists of central (2.5 mm annulus) and sub-perifoveal (5 mm annulus). Similar images analysis steps were used for deep choroidal *en face* images of both scan size except that the deep choroidal images were binarized using the Local Otsu method (square window size: 30 pixels for 3 x 3 mm and 15 pixels for 6 x 6 mm).[4] Figure 1 of the manuscript shows the modified ETDRS zone for the choriocapillaris and deep choroidal en face images of both scan sizes from which the indices were extracted.

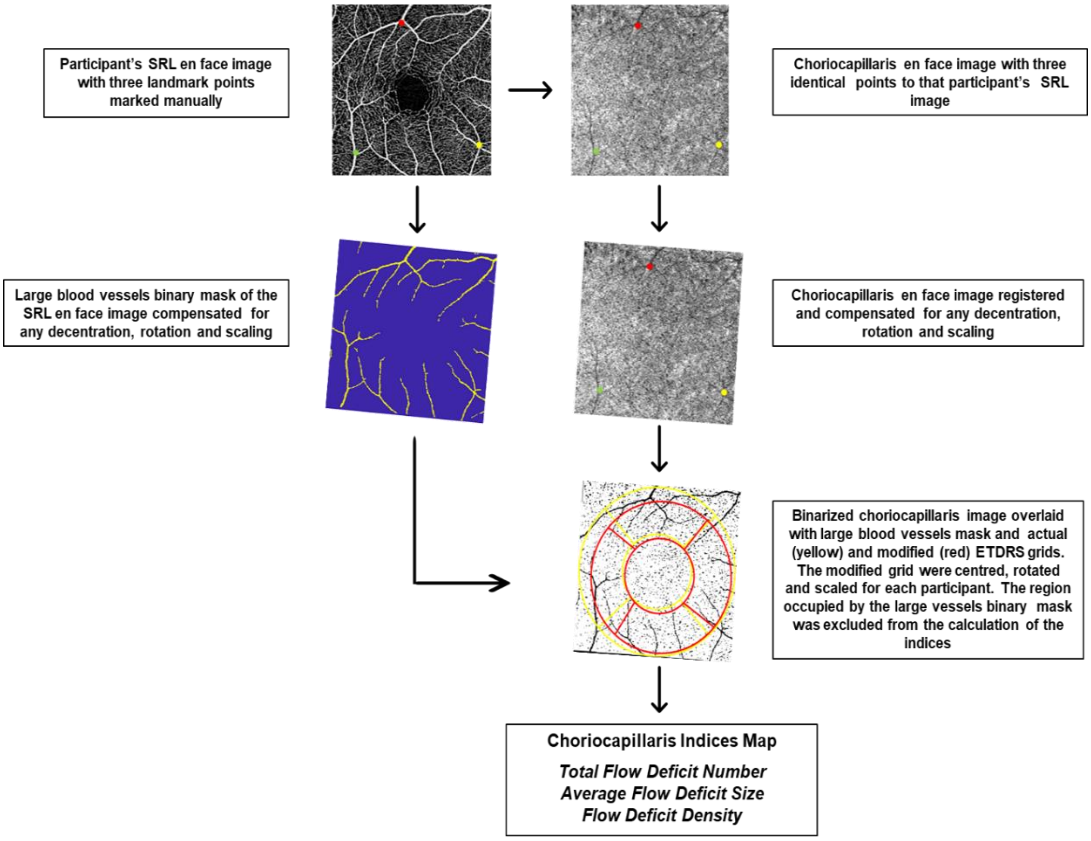

Figure S1. Overview of the image analysis process for extraction of indices for the choriocapillaris image from the 3×3 mm macular angiogram (SRL: superficial retinal layer, ETDRS: early treatment diabetic retinopathy study).

OCT scanning protocol, image analysis and choroidal thickness extraction

At each measurement session, two 5-line raster enhanced depth imaging (EDI) scan were captured using the Cirrus HD-OCT 5000 device, (Carl Zeiss Meditec Inc, Dublin, California, USA). Raw OCT data were exported in (.img) format from the OCT instrument and analysed using custom written semi-automated software in MATLAB (MathWorks, Massachusetts, United States).

The OCT-A images from the raster line centred on the fovea were analysed using previously described automated algorithm to segment outer boundary of retinal pigment epithelium (RPE) and the chorioscleral interface (CSI)[5]. All the images with their corresponding boundaries were manually corrected for any errors by a single experienced observer masked to the sessions. Image contrast was also optimized using Girard's contrast enhancement method[6] and the adaptive histogram equalization method to aid the visibility of the boundaries. The transverse scale of the OCT data was then adjusted to account for ocular magnification for each session and participant using the previously described schematic eye method[3] and were used to derive choroidal thickness (axial distance from the RPE to the CSI)[7] over the central 1 mm foveal zone, 1.5 mm parafoveal zone and 2.5 mm perifoveal zone.

Table S1. Amplitude of diurnal variation, acrophase, Rayleigh test results and repeated measures ANOVA (analysis of variance) outcomes for the choriocapillaris and deep choroidal OCT-A indices of the sub-parafoveal and sub-perifoveal quadrants for all participants. †P values from repeated measures ANOVA for time of day, refractive error, and time by refractive error. P<0.05 is highlighted in bold. SD: standard deviation, CD: circular deviation and df: degree of freedom.

Indices	Quadrant	Diurnal amplitude	Acrophase, Peak	Rayleigh test	Repeated measures ANOVA		
					Time of day (df=6, 258)	Time by refractive error (df=6, 252)	Refractive error (df=1, 42)
		Mean±SD	Time±CD (hours)	Rayleigh's r, P value	†P value		
Choriocapillaris-Flow Deficit Number	Parafovea-Superior	30.04±18.73	2:55AM±4.68	0.25,0.05	**0.04**	0.86	**<0.001**
	Parafovea-Nasal	32.45±19.06	3:32AM±4.60	**0.28,0.04**	0.11	0.65	**0.003**
	Parafovea-Inferior	28.05±16.65	2:25AM±4.02	**0.45,<0.001**	**0.003**	0.77	**0.001**
	Parafovea-Temporal	29.05±19.81	2:22AM±4.27	**0.37,0.003**	**0.002**	0.99	**0.001**
	Perifovea-Superior	65.39±34.97	5:29AM±4.81	0.21,0.14	0.08	0.64	**<0.001**
	Perifovea-Nasal	61.67±37.96	4:31AM±4.65	**0.26,0.049**	**0.04**	0.37	**<0.001**
	Perifovea-Inferior	61.13±41.16	4:49AM±4.82	0.20,0.14	0.40	0.45	**<0.001**
	Perifovea-Temporal	59.03±30.94	4:46AM±4.37	**0.35,0.003**	0.09	0.84	**<0.001**
Choriocapillaris-Average Flow Deficit Size (μm^2)	Parafovea-Superior	53.25±52.15	3:44AM±4.72	0.24,0.06	**0.03**	0.28	**<0.001**
	Parafovea-Nasal	64.71±82.49	2:37AM±4.59	**0.28,0.04**	0.28	**0.02**	**0.001**
	Parafovea-Inferior	41.71±36.45	2:19AM±4.61	**0.27,0.049**	0.14	0.10	**0.001**
	Parafovea-Temporal	67.34±71.45	3:09AM±4.33	**0.36,0.001**	0.07	0.05	**0.001**
	Perifovea-Superior	90.17±75.44	5:29AM±4.62	**0.27,0.049**	0.22	0.09	**0.001**
	Perifovea-Nasal	95.18±87.27	5:10AM±4.66	**0.27,0.049**	0.17	0.12	**0.002**
	Perifovea-Inferior	109.08±135.38	7:13AM±4.81	0.21,0.14	0.19	0.75	**0.002**
	Perifovea-Temporal	113.92±138.14	5:55AM±4.76	0.22,0.09	0.09	0.73	**0.001**
Choriocapillaris-Flow Deficit Density (%)	Parafovea-Superior	2.27±1.52	3:13AM±4.22	**0.39,0.001**	**0.001**	0.12	**<0.001**
	Parafovea-Nasal	2.62±2.03	3:15AM±4.51	**0.30,0.02**	**0.049**	**0.01**	**0.003**
	Parafovea-Inferior	1.88±1.23	2:42AM±4.03	**0.44,<0.001**	**0.004**	0.06	**0.002**
	Parafovea-Temporal	2.75±1.91	3:09AM±3.94	**0.47,<0.001**	**<0.001**	0.05	**0.001**
	Perifovea-Superior	2.40±2.07	5:47AM±4.55	**0.29,0.03**	0.06	0.26	**0.002**
	Perifovea-Nasal	2.52±1.95	4:33AM±4.35	**0.35,0.006**	**0.04**	0.23	**0.003**
	Perifovea-Inferior	2.43±2.21	6:14AM±4.84	0.20,0.14	0.09	0.81	**0.002**
	Perifovea-Temporal	2.61±2.43	5:01AM±4.58	**0.28,0.04**	**0.02**	0.69	**0.001**
Deep Choroidal-Perfusion Density (%)	Parafovea-Superior	1.77±1.04	5:01AM±4.82	0.20,0.14	0.33	**0.01**	0.61
	Parafovea-Nasal	1.96±1.44	4:50AM±4.10	**0.42,<0.001**	**0.02**	0.69	0.66
	Parafovea-Inferior	1.86±1.01	3:46AM±4.30	**0.37,0.001**	**0.049**	0.28	0.20
	Parafovea-Temporal	1.79±1.01	5:56AM±4.63	**0.27,0.049**	**0.04**	**0.02**	0.24
	Perifovea-Superior	1.77±1.10	3:33AM±4.86	0.19,0.14	**0.003**	0.28	0.61
	Perifovea-Nasal	1.56±0.89	3:19AM±4.15	**0.41,0.001**	**0.001**	0.75	0.12
	Perifovea-Inferior	1.79±1.03	2:02AM±4.51	**0.30,0.03**	**0.008**	0.70	0.84
	Perifovea-Temporal	1.96±1.17	4:08AM±4.40	**0.34,0.005**	**<0.001**	0.21	0.11

Table S2: Daily mean (±SD (standard deviation)) outcomes for the choriocapillaris and deep choroidal OCT-A indices of the sub-foveal, sub-parafoveal and sub-perifoveal regions for all participants, myopes and non-myopes.

Indices	Sub-macular regions	Total	Myopes	Non-myopes
		Mean±SD	Mean±SD	Mean±SD
Choriocapillaris- Total Flow Deficit Number	Fovea	272.33±42.88	289.33±44.92	251.93±29.47
	Parafovea	1446.76±220.77	1543.10±222.04	1337.74±158.99
	Perifovea	2499.66±426.47	2694.80±418.69	2265.49±299.89
	Parafovea-Superior	363.24±55.52	387.66±55.31	333.93±39.33
	Parafovea-Nasal	358.66±57.49	380.22±59.71	332.78±42.70
	Parafovea-Inferior	360.93±56.76	385.03±56.71	332.02±41.34
	Parafovea-Temporal	366.93±54.86	390.19±54.33	339.02±40.80
	Perifovea-Superior	617.00±107.75	665.57±106.90	558.72±75.14
	Perifovea-Nasal	630.54±115.06	681.71±110.85	569.13±86.74
	Perifovea-Inferior	616.32±113.16	667.16±111.13	555.31±81.09
	Perifovea-Temporal	635.80±98.75	680.35±97.94	582.33±68.85
Choriocapillaris- Average Flow Deficit Size (μm^2)	Fovea	399.26±189.52	481.18±217.03	302.36±76.89
	Parafovea	374.05±172.29	449.93±195.66	283.01±69.55
	Perifovea	608.53±275.43	726.76±317.73	466.65±103.15
	Parafovea-Superior	372.05±169.94	451.11±190.33	277.13±64.20
	Parafovea-Nasal	377.67±186.01	456.93±213.07	282.56±76.77
	Parafovea-Inferior	359.77±162.36	428.82±186.34	276.91±66.15
	Parafovea-Temporal	386.72±176.80	462.80±200.35	295.43±77.88
	Perifovea-Superior	582.92±190.94	659.94±223.64	490.49±74.03
	Perifovea-Nasal	601.75±272.65	714.74±316.30	466.16±107.94
	Perifovea-Inferior	619.08±297.02	742.92±345.62	470.49±111.56
	Parafovea-Temporal	623.86±273.42	741.69±313.73	482.46±107.04
Choriocapillaris- Flow Deficit Density (%)	Fovea	14.15±6.72	16.93±7.50	10.81±3.38
	Parafovea	13.50±6.44	16.22±7.22	10.23±3.05
	Perifovea	10.70±5.92	12.91±6.30	8.04±2.59
	Parafovea-Superior	13.46±6.42	16.35±7.11	9.99±2.84
	Parafovea-Nasal	13.50±6.93	16.27±7.86	10.19±3.42
	Parafovea-Inferior	12.95±6.13	15.46±6.94	9.93±2.89
	Parafovea-Temporal	14.07±6.58	16.80±7.33	10.80±3.38
	Perifovea-Superior	10.25±5.40	12.46±6.17	7.61±2.41
	Perifovea-Nasal	10.72±5.67	12.88±6.47	8.12±2.90
	Perifovea-Inferior	10.76±5.99	13.10±6.86	7.95±2.83
	Parafovea-Temporal	11.07±5.34	13.22±6.07	8.49±2.53
Deep Choroidal- Perfusion Density (%)	Fovea	57.22±4.41	56.89±4.51	57.61±4.26
	Parafovea	53.98±3.68	53.54±3.44	54.50±3.90
	Perifovea	51.57±2.79	51.14±2.39	52.10±3.13
	Parafovea-Superior	54.40±3.99	54.18±3.98	54.66±3.99
	Parafovea-Nasal	55.15±4.00	54.94±3.91	55.41±4.11
	Parafovea-Inferior	52.93±3.66	52.33±3.33	53.66±3.91
	Parafovea-Temporal	53.42±4.69	52.72±4.51	54.26±4.78
	Perifovea-Superior	51.29±3.19	51.12±2.37	51.50±3.96
	Perifovea-Nasal	54.00±3.70	53.24±3.59	54.92±3.64
	Perifovea-Inferior	49.80±3.08	49.74±2.97	49.87±3.22
	Parafovea-Temporal	51.20±3.70	50.46±3.65	52.10±3.58

References

1 Lin, E. *et al.* Are choriocapillaris flow void features robust to diurnal variations? A swept-source optical coherence tomography angiography (OCTA) study. *Sci Rep* **10**, 1-9, doi:10.1038/s41598-020-68204-x (2020).

2 Chu, Z., Zhang, Q., Gregori, G., Rosenfeld, P. J. & Wang, R. K. Guidelines for imaging the choriocapillaris using OCT angiography. *Am J Ophthalmol* **222**, 92-101, doi:10.1016/j.ajo.2020.08.045 (2021).

3 Lal, B., Alonso-Caneiro, D., Read, S. A. & Carkeet, A. Induced refractive error changes the optical coherence tomography angiography transverse magnification and vascular indices. *Am J Ophthalmol* **229**, 230-241, doi:10.1016/j.ajo.2021.04.012 (2021).

4 Siegfried, F. *et al.* Evaluating diurnal changes in choroidal sublayer perfusion using optical coherence tomography angiography. *Acta Ophthalmol*, doi:10.1111/aos.14140 (2019).

5 Alonso-Caneiro, D., Read, S. A. & Collins, M. J. Automatic segmentation of choroidal thickness in optical coherence tomography. *Biomed Opt Express* **4**, 2795-2812, doi:10.1364/BOE.4.002795 (2013).

6 Girard, M. J., Strouthidis, N. G., Ethier, C. R. & Mari, J. M. Shadow removal and contrast enhancement in optical coherence tomography images of the human optic nerve head. *Invest Ophthalmol Vis Sci* **52**, 7738-7748, doi:10.1167/iovs.10-6925 (2011).

7 Rosenfeld, P. J. *et al.* ZEISS Angioplex spectral domain optical coherence tomography angiography: Technical aspects. *Dev Ophthalmol* **56**, 18-29, doi:10.1159/000442773 (2016).
